# Human ancestry indentification under resource constraints -- what can one chromosome tell us about human biogeographical ancestry?

**DOI:** 10.1186/s12920-018-0412-4

**Published:** 2018-11-20

**Authors:** Tanjin T. Toma, Jeremy M. Dawson, Donald A. Adjeroh

**Affiliations:** Lane Department of Computer Science and Electrical Engineering, West Virginia University, Morgantown, WV USA

**Keywords:** SNP, DNA, Ancestry prediction, Single chromosome

## Abstract

**Background:**

While continental level ancestry is relatively simple using genomic information, distinguishing between individuals from closely associated sub-populations (e.g., from the same continent) is still a difficult challenge.

**Methods:**

We study the problem of predicting human biogeographical ancestry from genomic data under resource constraints. In particular, we focus on the case where the analysis is constrained to using single nucleotide polymorphisms (SNPs) from just one chromosome. We propose methods to construct such ancestry informative SNP panels using correlation-based and outlier-based methods.

**Results:**

We accessed the performance of the proposed SNP panels derived from just one chromosome, using data from the 1000 Genome Project, Phase 3. For continental-level ancestry classification, we achieved an overall classification rate of 96.75% using 206 single nucleotide polymorphisms (SNPs). For sub-population level ancestry prediction, we achieved an average pairwise binary classification rates as follows: subpopulations in Europe: 76.6% (58 SNPs); Africa: 87.02% (87 SNPs); East Asia: 73.30% (68 SNPs); South Asia: 81.14% (75 SNPs); America: 85.85% (68 SNPs).

**Conclusion:**

Our results demonstrate that one single chromosome (in particular, Chromosome 1), if carefully analyzed, could hold enough information for accurate prediction of human biogeographical ancestry. This has significant implications in terms of the computational resources required for analysis of ancestry, and in the applications of such analyses, such as in studies of genetic diseases, forensics, and soft biometrics.

## Background

Accurate inference of biogeographical ancestry is important for various application areas. For instance, population stratification can confound the relationship between a genetic marker and disease. Identifying ancestry informative markers (AIMs) in the genome is essential for detecting such stratification in case-control association studies of complex diseases, such as cancer, diabetes, neurodegenerative diseases (e.g., Alzheimer’s disease), and cardiovascular diseases [1–3]. Measuring genetic ancestry has also been a focus in the forensic science community. For routine forensic identification of ancestry, a small number of genetic markers is needed that can be tested quickly and cheaply [4, 5]. Reliable estimation of biogeographic ancestry is also a key procedure in studies of admixed populations. Several AIM sets have been proposed for estimating the admixture between given ancestral populations, for insance, the genetic contributions of Africans and Europeans to African American populations, and the contributions of Native Americans and Africans or African Americans to Latino populations [6–8]. Ancestry estimation also plays a significant role in guiding criminal investigations [9, 10]. Furthermore, many studies are investigating the association between ancestry and certain types of diseases [11–13]. Thus, analysis of genetic ancestry is a vast research area with numerous applications, which has attracted the use of a diverse array of techniques.

One aim in studies on human genetic ancestry is to identify sets of ancestry informative markers (AIMs) by analyzing DNA sequences from different chromosomes collected from the population samples under study. Most widely used AIMs are based on single nucleotide polymorphisms (SNPs) [14, 15] which demonstrate superior ability in predicting biogeographical origin of an unknown individual compared to other markers, such as short tandem repeats (STRs) [16]. Although a large number of SNPs can provide nearly accurate ancestry information for multiple geographic regions, a small but robust set of SNPs may be more desirable for certain applications [17]. Several published SNP panels have focused on distinguishing ancestral origins for individuals from different continental regions, e.g., Europe, America, Africa and East Asia [18], or between people from widely-separated global populations [1]. Some also proposed small SNP panels, typically ranging from the teens to hundreds of SNPs, which can estimate continental genetic ancestry relatively well [18]. However, very few studies have focused on identifying SNP panels for sub-continental ancestry estimation, a known challenging problem, given the difficulties of using small SNP panels in distinguishing individuals from closely related populations [19].

Several studies on ancestry identification have demonstrated that many globally distributed populations, can generally be distinguished by examining differences in allele frequencies, using the fixation index, widely known as F_st_ [20]. These studies identified that thousands of single nucleotide polymorphisms (SNPs) distributed throughout the human genome have significant differences in allele frequencies between two or more continental populations [21, 22]. Thus, a small set of SNPs (e.g., a few hundred) can be used to separate individuals with different continental origins using the F_st_ feature [23–25]. However, such panels of SNPs are less informative in detecting sub-continental differences in closely related populations [6, 19, 26–30]. Apart from F_st_ based ancestry estimation, techniques based on principal component analysis (PCA) [31–33] have widespread applications. A typical example of this class of methods is EIGENSTART [31]. These methods represent genetic variations by principal component vectors, however, they are not highly efficient due to the need for a large number of SNPs (thousands to millions) to calculate the principal component vectors. Besides, many studies have developed small panels of SNPs to distinguish ancestral origins from a large number of populations, example, 73 populations (Kidd et al. [34]) and 119 populations (Kidd et al. [17]). However, they used unsupervised learning (clustering) methods, such as STRUCTURE [35], to determine which populations cluster together and thus observed the ability of a SNP panel to infer ancestry.

Important progress has been made in the use of genomic information for ancestry detection [36–39], however, significant challenges still remain. Although a panel with a small number of SNPs can produce sufficiently accurate continental-level ancestry classification, reliable sub-continental population detection using only limited number of marker SNPs is still a major challenge. Significant research is still required to identify sets of ancestry informative SNPs (AISNPs) that can accurately distinguish closely related sub-populations, e.g., those from the same continent. This is a difficult multi-class classification challenge, with only a few attempts at the problem. This problem is also related to the issue of separating admixture populations [7, 8, 40, 41], and recent approaches that have used GWAS (Genome-Wide Association Studies) data [2, 3, 41]. We do not address the problem of admixture in the current paper, and we do not use GWAS datasets.

Another significant challenge is that of computation, and the ever limited resources available in most labs, where such ancestry estimation or classification may be needed. Thus, given resource limitations, we introduce a key new constraint in addressing the problem: only SNPs from one chromosome can be used in the analysis. This is significant, as it means that the sequencing needed can be focused on only the specified chromosome, hence saving time and sequencing cost. Essentially, the challenge, therefore, is to answer the question: how much information about our human biological and geographical ancestry can we found in a single chromosome? Clearly, this question can be formulated at different levels of granularity, for instance, using sets of chromosomes, rather than just one chromosome, or using sets of genes, rather than chromosomes.

In this work, we address the problems of both continental-level and sub-continental level ancestry identification using small SNP panels, with all SNPs in the panel coming from just one single chromosome. For this study, we will focus on Chromosome 1, since this is the largest chromosome, and thus might provide the best starting point for our exercise. Thus, in this work, we have employed machine learning approaches and statistical methods to determine small sets of SNPs that can be used to predict an individual’s biogeographical origin to continental as well as sub-continental levels. Here, we studied DNA information from Chromosome 1 (largest human chromosome) to develop an efficient and cost-effective ancestry inference system.

We consider the problem in three stages. Initially, we employed parameter-based SNP selection, and later refined the selection by using a clustering technique (specifically, DBSCAN [42, 43]) to choose an efficient panel of SNPs. The final SNP panel is selected by applying a statistical approach based on pairwise correlation of SNPs to identify important ancestry informative SNPs for both continental and sub-continental ancestry classification. For continental-level ancestry classification, we view it as a five-class classification problem including the continents of Europe, Latin America, Africa, East Asia, and South Asia. Within each continent, we also have several closely-related sub-populations. Distinguishing these sub-populations accurately is the challenging part. To address the sub-continental classification problems, we consider pairwise classification of the sub-populations within each continent. For both continental and sub-continental classification problem, we have applied the softmax neural network classifier [44].

## Methods

**Fig. 1:**
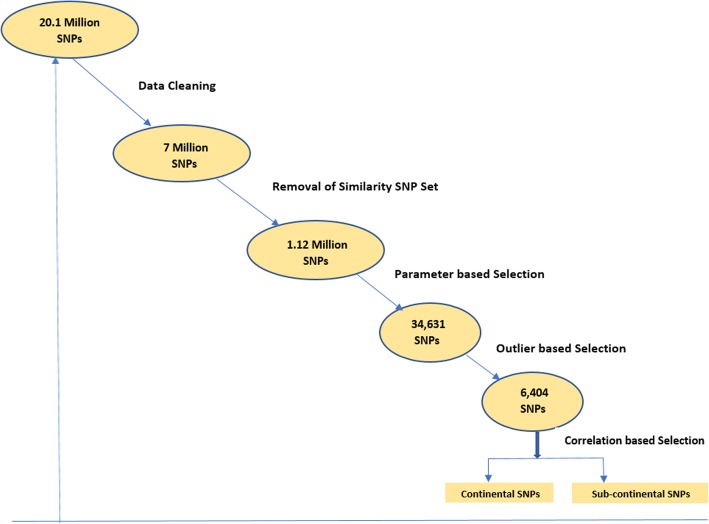
Graphical depiction of the proposed process of SNP selection for predicting human biogeographical ancestry

Figure 1 shows a schematic diagram of the proposed process for selection of ancestry informative SNPs. The figure shows how the initial set of over 20 million SNPs from chromosome 1 is reduced in several data pre-processing stages (e.g., data cleaning, similarity SNP set removal), and initial pruning stages (parameter-based selection and outlier-based selection) to a much smaller set of 6404 SNPs. Below, we describe our methodology in more detail.

### Datasets and pre-processing

In this study, we used datasets from the 1000 Genome Project, Phase 3 [19]. The dataset contains information from 2504 individuals, from 26 different sub-populations, spanning five continents. For each individual, information is provided on 84.4 million variants (SNPs) from all 23 chromosomes. Table 1 provides a summary on the different populations, including the number of samples in each of the 26 populations. We analyzed the variants from only Chromosome 1 which is nearly 20.1 million SNPs. After the pre-processing steps (e.g., data cleaning), we identified continental and sub-continental ancestry informative SNPs in several stages. The DNA information for the 20.1 million variants (SNPs) from Chromosome 1 of each of the 2504 subjects is provided in a large 61.2 GB .vcf file. At the beginning, we extracted data from the .vcf file and stored them in several .mat files to be able to conduct our analysis in a MATLAB (MathWorks Inc., Natick, MA) environment. For each SNP, we extracted their rsID, loci number/position, reference allele, alternate allele(s), and allele information of all 2504 subjects (each person’s allele is diploid, containing two nucleotides, from different combinations of the four nucleotide bases A, C, G, T). Next, we performed data cleaning operations on the extracted data based on the following criteria:

- Remove an SNP position if the SNP contains more than one reference nucleotides.
- Exclude an SNP position in the analysis, if an alternate allele nucleotide also exists in the reference allele for this SNP,
- Exclude an SNP locus from the analysis, if for the given SNP, each of the two nucleotides from all the individuals in the dataset both match with the reference allele’s nucleotide.

**Table 1:** 26 populations in the dataset

Population code	Population name	Continent	Sample size
PUR	Puerto Rican	America	104
CLM	Colombian	America	94
PEL	Peruvian	America	85
MXL	Mexican-American	America	64
GBR	British	Europe	91
FIN	Finnish	Europe	99
IBS	Spanish	Europe	107
CEU	CEPH	Europe	99
TSI	Tuscan	Europe	107
CHS	Southern Han Chinese	East Asia	105
CDX	Dai Chinese	East Asia	93
KHV	Kinh Vietnamese	East Asia	99
CHB	Han Chinese	East Asia	103
JPT	Japanese	East Asia	104
PJL	Punjabi	South Asia	96
BEB	Bengali	South Asia	86
STU	Sri Lankan	South Asia	102
ITU	Indian	South Asia	102
GIH	Gujarati	South Asia	103
ACB	African-Caribbean	Africa	96
GWD	Gambian	Africa	113
ESN	Esan	Africa	99
MSL	Mende	Africa	85
YRI	Yoruba	Africa	108
LWK	Luhya	Africa	99
ASW	African-American SW	Africa	61

The result of the above is the removal of around 13 million SNPs at the cleaning stage. Further analysis is then performed on the remaining SNPs. For the purpose of SNP selection, we removed a person’s allele information from an SNP position, if the person’s two nucleotides at the given position are the same as the reference allele’s nucleotide. Consequently, two different sets of SNPs have been observed in the analysis. In one set, each SNP contains same allele information among all individuals, although this allele information is different from the reference nucleotide. We call this SNP set the ‘Similarity set’. In contrast, in the other set, allele information is not the same among all individuals at the given SNP position. We call this set the ‘Dissimilarity set’. Since, for ancestry identification, we need to distinguish among populations with respect to some attribute/feature(s), SNP loci that demonstrate greater variation in DNA information among individuals will lead to better identification results. Thus, we have chosen only the ‘Dissimilarity set’ of SNPs for further analysis.

### SNP selection

The overall process of SNP selection is performed in three stages, each building on the results from the preceding stage. The initial stage employs a parameter-based selection; the latter stages use machine learning and statistical methods to further improve the results, and to prune the selected SNPs to significantly smaller set.

1. Stage 1: Parameter-based SNP Selection:

At the beginning, we aimed to identify important markers for each of the 26 populations from the ‘Dissimilarity set’ of SNPs. Consequently, we generated a structure array where each row allocates information from one SNP position containing 26 different fields, with each field corresponding to one of the 26 different populations. Each field associated with one population group contains relevant information regarding that group, such as, number of individuals of that group existing at that SNP position (since we removed individuals from a SNP position based on the similarity of their allele with reference nucleotide) and corresponding allele information of those individuals. Next, we calculated two parameters ‘α’ and ‘β’ at each dissimilar SNP position for each of the 26 populations using the following formulae:

$$ \alpha =\frac{n_p^i}{n_p}\kern0.5em \mathrm{and}\kern0.5em \beta =\frac{f_p^i}{n_p^i}, $$

where, *p =* 1, 2, …, 26

${n}_p^i$= No. of individuals of population type *p* existing at SNP *i*

*n*_*p*_= Total no. of individuals of population *p* in training data

${f}_p^i$= Frequency of occurrence of the allele that appears most in population *p* at SNP *i*

For a given population *p*, a SNP position *i* is considered important if at that position α × β = 1 (i.e., α = 1 and β = 1). Here, α = 1 indicates that all individuals of that population exist at SNP *i*, since none of them has both nucleotides being the same as the reference nucleotide, while β = 1 means those individuals also share the same allele information at SNP *i*. Thus, based on the values of parameters α and β, we identify the best distinguishing SNPs for each population. After we obtain important SNP sets for each population, we take the union of all the 26 sets. The result is a set of 38,532 ancestry informative SNPs. From these 38 K SNPs, we further removed the SNPs which contain the same allele information across all individuals from all 26 populations in the training set, since SNPs showing no variations between different population groups are not informative in distinguishing them. At the end of this stage, the result is a set of 34,631 ancestry informative SNPs, all from Chromosome 1.

2. Stage 2: Outlier-Based SNP Selection:

In order to reduce the number of SNPs further, we apply a cluster-based technique on the results from Stage 1. In particular, we use a contrarian approach: we group the SNPs using a clustering technique. In doing so, we also indirectly identify those SNPs that could not be grouped comfortably into any particular cluster. These are the outlier SNPs that do not seem to be similar with other SNPs, and thus represent good candidates for use in discriminating between ancestries. We use DBSCAN [42, 43] as the clustering technique for further selection of important AISNPs which are reasonably distinct in nature. This is a density-based clustering technique which does not require the number of clusters of the data to be pre-specified. Given a set of data points in some space, the DBSCAN clustering approach attempts to place points that are closely packed into one group. Points that lie alone in low-density regions are marked as outliers. In our specific problem of ancestry classification, SNPs that contain similar ancestry information are clustered together, while those that could not be clustered into some group are identified as outliers with seemingly unique ancestry information. In this work, we have considered these outlier SNPs as good candidates for distinguishing biogeographical ancestry between populations.

Here, we apply DBSCAN clustering on the 34,631 SNPs extracted in the previous stage of selection. The algorithm requires three input parameters, namely, data matrix *D*, radius parameter (ε) and neighborhood density threshold (*MinPts*). Data matrix *D* has 34,631 rows, where each row is associated with one SNP. Each SNP is considered as an object with *l* dimensions, where *l* is the number of training samples. Each dimension belongs to the allele information of a training subject represented by a number between 1 and 16, since four nucleotides {A, C, G, T} generate 16 possible allele symbols {AA, AC, …, TT}. The parameter *MinPts* (neighborhood density threshold) indicates the minimum number of points required to form a cluster, while ε (the radius parameter) is measured as the Euclidean distance between two *l*-dimensional SNP objects. The DBSCAN clustering algorithm is described in Algorithm 1 below using a pseudo code [43].

The choice of the two parameters, ε and *MinPts,* requires careful consideration as they play important roles in determining the output clusters. For this problem, we have set MinPts = 2, i.e., at least two SNPs will be able to form a cluster if they are within a certain distance ε. And, the value of ε is chosen empirically. We measured the 26-class classification performance for different values of ε for the 80/20 train-test split of the data. For *ε*=0.1 we obtained the best classification result. The DBSCAN clustering technique resulted in 2378 clusters and 6404 outliers. These 6404 outlier SNPs constitute our new set of candidate SNPs for ancestry identification.

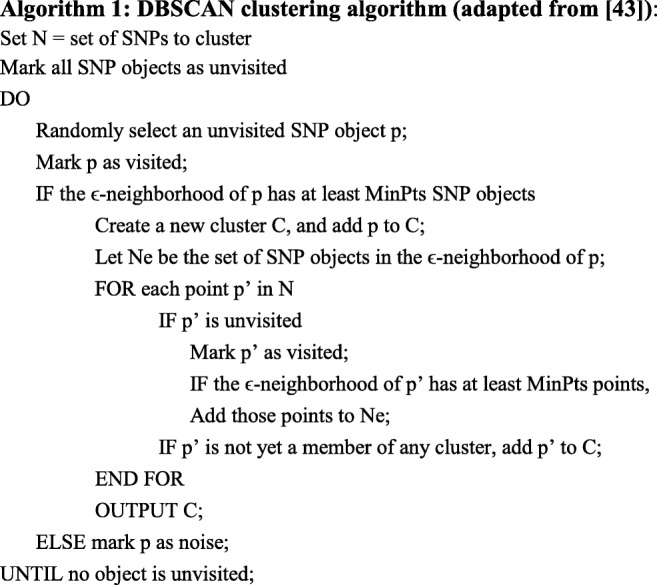

3. Step 3: Correlation-based SNP Selection:

As we obtain the set of 6404 SNPs from the clustering technique, we measure the overall 26-class ancestry prediction performance for each individual SNP marker. That is, we perform ancestry estimation using each of the 6404 SNPs, independent of the other SNPs. Naturally, we do not expect to produce very good performance for a single SNP. However, the relative performance of the SNPs is a crucial piece of information for our approach. Consequently, we generate a performance matrix *X* with *m*=6404 rows, where each row of *X* is allocated for one SNP representing a six-dimensional vector,

$$ {\underline{x}}^{(i)}=\left[{x_1}^{(i)}\;{x_2}^{(i)}\;{x_3}^{(i)}\;{x_4}^{(i)}\;{x_5}^{(i)}\;{x_6}^{(i)}\right] $$

The first element records the accuracy of 26-class classification using SNP *i*. The next five elements of the vector are related to five continents, where each element denotes the percentage of test individuals correctly predicted from a continent. Classification into 26 populations by each SNP has been conducted using an 80–20% train-test split, with *n* = 2504 individuals. For classification, the SNP is represented using its allele-context feature, where each SNP’s allele-context feature belongs to three possible values: 0, 1, 2. For the allele-context feature, a ‘0’ means both nucleotides from a person are the same as the reference nucleotide, ‘1’ means one of the two nucleotides is different from the reference nucleotide, and ‘2’ means both nucleotides of that person are different from the reference nucleotide at SNP *i*. For both the training sets and test sets, we denote the allele-context feature vector *a* and class-label vector *b* as follows:

$$ {\underline{a}}_{train}^{(i)}={\left[{a}_1^{(i)}{a}_2^{(i)}..\dots {a}_l^{(i)}\right]}^T\;\mathrm{and}\kern0.5em {\underline{a}}_{test}^{(i)}={\left[{a}_1^{(i)}{a}_2^{(i)}..\dots {a}_{\left(n-l\right)}^{(i)}\right]}^T $$

$$ {b}_{train}={\left[{b}_1\;{b}_2..\dots {b}_l\right]}^T\kern0.5em \mathrm{and}\kern0.5em {b}_{test}={\left[{b}_1\;{b}_2..\dots {b}_{\left(n-l\right)}\right]}^T $$

Here, *l* = number of training subjects, and *n-l* = number of test subjects. Thus, for *i* = 1,2, …, *m* number of SNPs, the overall performance matrix is represented as,

$$ X={\left[{\underline{x}}^{(1)}{\underline{x}}^{(2)}..\dots {\underline{x}}^{(6404)}\right]}^T $$

Having created the performance matrix *X*, we can now compute the pairwise correlation between the SNPs using the associated performance vectors. For example, correlation of SNP *i* and SNP *k* is calculated using the Pearson’s correlation coefficient as follows:

$$ C=\frac{\sum \limits_{j=1}^5\left({x}_j^{(i)}-{x}^{-(i)}\right)\;\left({x}_j^{(k)}-{x}^{-(k)}\right)}{\sqrt{\sum \limits_{j=1}^5{\left({x}_j^{(i)}-{x}^{-(i)}\right)}^2}\kern0.5em \sqrt{\sum \limits_{j=1}^5{\left({x}_j^{(k)}-{x}^{-(k)}\right)}^2}} $$

Here,

${x}_j^{(i)}$=element of the vector ${\underline{x}}^{(i)}$ for continent *j* (*j* = 1,2,..,5),

*x*^−(*i*)^=average of the five ${x}_j^{(i)}$elements of vector ${\underline{x}}^{(i)}$.

Now, if SNP *i* and SNP *k* are highly correlated (that is, their correlation coefficient *C* is above a certain threshold *th*), then one of them is kept in the analysis and the other one is removed. Here, we consider the SNP that provides a better classification accuracy in the performance matrix (represented by the first element of vector ${\underline{x}}^{(i)}$) as “non-redundant”, while the other SNP is taken to be redundant. The proposed correlation-based approach to SNPs selection is explained in more detail below, using pseudo code (see Algorithm 2).

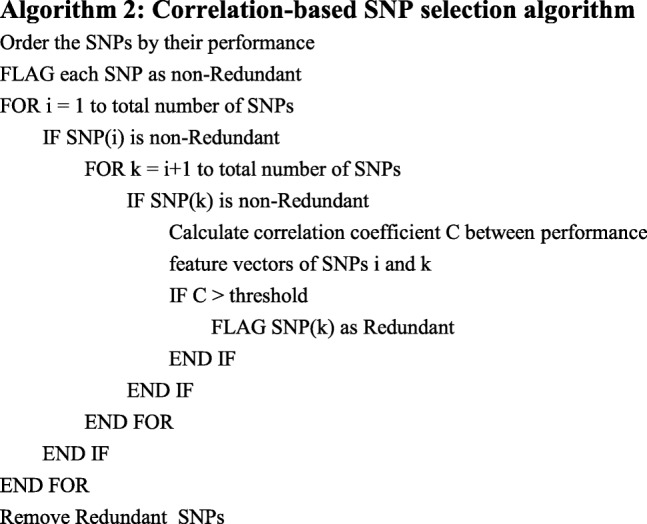



Now that we have presented the general procedure for selecting the SNPs, the final step will be to select those that are best for continental-level classification, and those that are more suitable for more localized discrimination between sub-populations, say from the same continent. We describe our approach below:

- SNP selection for continental-level classification

To determine the best candidate SNPs for continental-level classification, we have exploited the proposed correlation-based SNP selection. First, the 6404 SNPs are ranked from highest to lowest based on their classification accuracy in the performance matrix *X* and the 6404 × 6 performance matrix is rearranged accordingly. Following this ranking, we create the ordered listing of the SNPs for the initial ‘non-Redundant SNP set’ and the algorithm is then initialized with the best performing SNP. For a certain correlation threshold *th*, the algorithm is executed to identify the final set of non-Redundant SNPs from the 6404 SNPs. These candidate SNPs represented by the allele-context feature are subsequently used to perform the five-continent classification using an 80/20 train-test split. We carried out empirical experiments for a range of values of correlation thresholds and the threshold which provides the best classification performance with the smallest set of SNPs has been finally selected.

b) SNP selection for pairwise/binary classification between sub-populations

Having determined the continental-level ancestry using the above, the next question is how to differentiate two sub-populations, within the same continent. When an individual’s continental ancestry is known and the individual belongs to any of two possible closely related sub-populations within that continent, the issue now becomes how to identify the accurate sub-population ancestry. In this work, we have selected candidate SNP sets for all possible pairwise classification of sub-populations within a given continent exploiting the same basic correlation-based SNP selection algorithm used for continental-level ancestry identification. Given two sub-populations, say *S*_1_ and *S*_2_ from the same continent *j,* the goal is to identify a powerful set of candidate SNPs which will be able to distinguish individuals from these two populations. Now, the 6404 SNPs are ranked from highest to lowest based on the continent *j* elements ${x}_j^{(i)}$ in the performance matrix X and performance matrix is rearranged accordingly. Thus, the correlation algorithm is initialized with the best performing SNP for continent *j* and for a certain threshold the algorithm is executed to obtain the required set of SNPs from the 6404 SNPs. Next, we perform binary classification between the two sub-populations using the allele-context feature of these SNPs, again following an 80/20 train-test split. As was done for the continental-level classification, we also tested for a different values of the correlation threshold, and selected the threshold that provided the best classification performance while using a small number of SNPs.

### Ancestry classification using selected SNPs

Having identified the best SNP subsets, ancestry classification can be performed using standard classification algorithms. In this work, we perform classification using the softmax neural network classifier [44]. We use the same algorithm for both continental-level classification and for sub-population-level classification. In machine learning, softmax regression is a generalization of binary logistic regression that we can use for multi-class classification tasks. In logistic regression, the output labels are assumed to be binary, that is, *y*^(*i*)^ ∈ {0, 1}. The goal then is to predict the probability that a given sample belongs to the ‘1’ class, i.e., *P*(*y* = 1|*x*) vs. the probability that it belongs to the ‘0’ class, i.e., *P*(*y* = 0|*x*). On the other hand, in softmax regression setting, the output label can take *K* different values: *y*^(*i*)^ ∈ {1, 2⋯, *K*}. Now, the goal is to estimate the probability for each value *k* ∈ {1, 2⋯, *K*}, i.e., *P*(*y* = *k*|*x*). Thus, softmax regression is an extension of logistic regression to the multi-class case. With *K* = 2, softmax regression is same as binary logistic regression. Overall, with softmax regression scheme, we can solve the classification problem not just for *K* = 2, but also for many possible values of *K*.

Softmax regression is often used as the activation function in the final layer of a neural network classifier. For a *K*-class classification problem, the number of units/nodes in the output layer of the neural network should be *K*. Each of the *K* output nodes gives the probability of a certain class and probabilities from all output nodes sum to 1. Each output node *i* in the final layer of the neural network receives the weighted sum of the inputs from the previous layer with the addition of a bias term, which is denoted as follows,

$$ {z}_i=\sum \limits_j{w}_{i,j}{x}_j+{b}_i $$

where, *j* is the number of nodes in the previous layer. Now to compute the softmax activation at each output node, exponential of the term *z*_*i*_ is calculated for each *i*,

$$ {t}_i={e}^{z_i} $$

Finally, activation at output node *i* is obtained by normalizing the exponential term.

$$ {a}_i={t}_i/{\sum}_{i=1}^K{t}_i $$

Thus, by normalizing the distribution, output from each node *i* falls in the range [0, 1]. Here, the class associated with the highest probability value is considered as the predicted output label.

## Results

Experiments were performed using the identified 1000 Genome dataset, with 26 sub-populations, from 5 continents. The performance of the proposed approach was evaluated, on both continental-level and sub-population ancestry prediction/ classification. The results of these experiments are described below.

### Continental classification

**Fig. 2:**
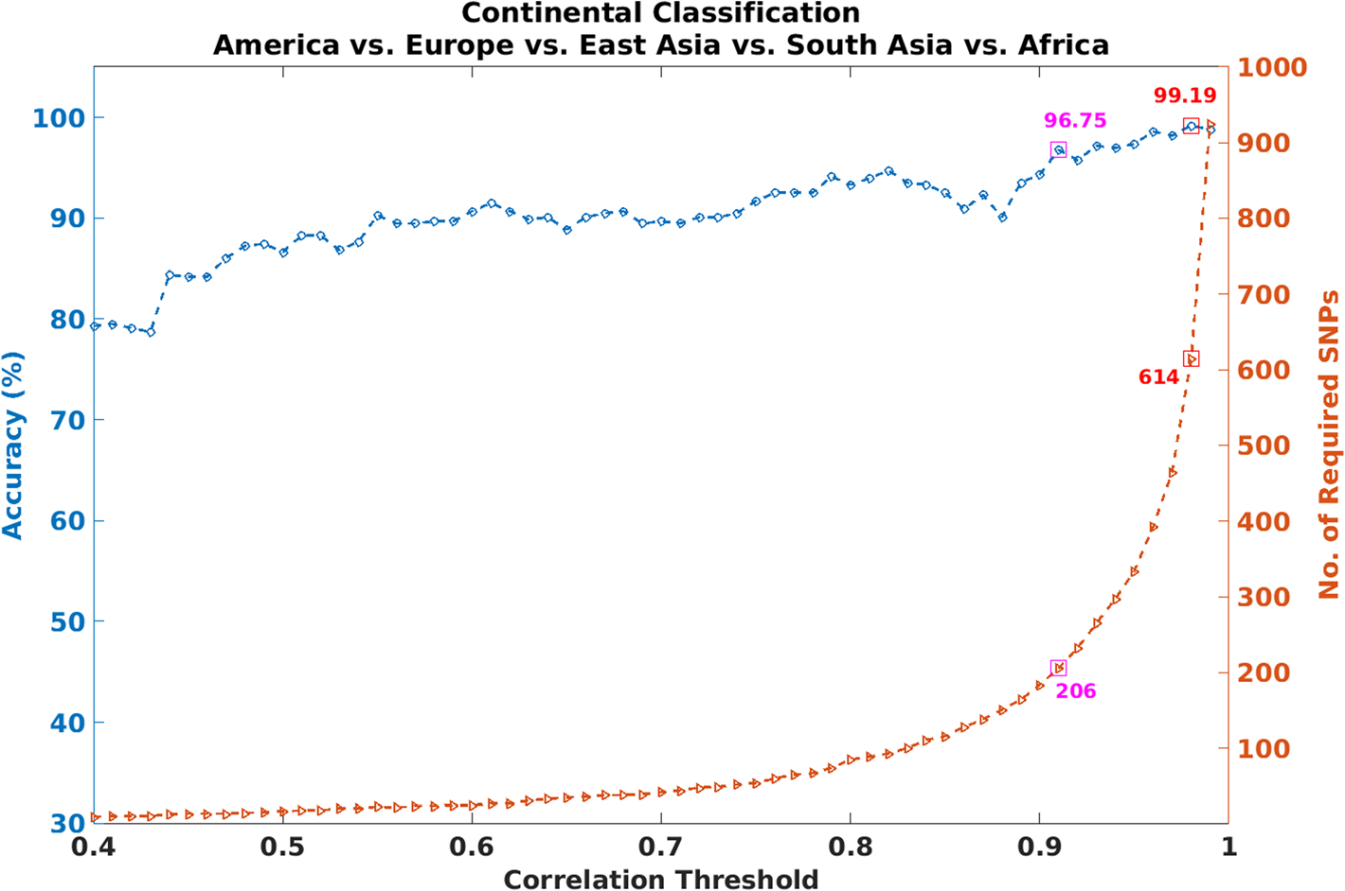
Results for continental-level ancestry classification using varying thresholds. Results include both accuracy (left) and the number of SNPs (right) required to achieve a given accuracy

First, we performed a five-class classification (using the five continents -- Europe, America, East Asia, South Asia, and Africa) for a range of values of the correlation threshold: *th* = 0.1 to 0.99 with an interval of 0.01. In Fig. 2, we show the results on continental-level classification for correlation threshold *th* = 0.4 to 0.99 with 0.01 interval along with the corresponding number of SNPs. The highest performance achieved is 99.91% for *th* = 0.98 with 614 SNPs (marked by a red square in the plot). But, since our goal is to rather use a smaller panel of SNPs to distinguish the continental populations, we searched for the threshold *th* that provides an optimum performance with less number of SNPs (approximately 200 or less). From Fig. 2, we can observe the general trend in performance for the proposed approach. At *th* = 0.7, the system suggests a panel of 32 SNPs, for an overall classification accuracy of about 90%. Performance generally increases with increasing correlation threshold, rising to about 93% accuracy rate, at about *th* = 0.83, using about 93 SNPs. The best classification result is obtained with correlation threshold *th* = 0.91, resulting in a classification accuracy of 96.75% with 206 SNPs (marked by the magenta square). These 206 SNPs have been considered as our final candidate SNPs for continental-level ancestry classification. The confusion matrix for the five-class classification problem with overall performance of 96.75 96.75% is shown in Table 2. With respect to each continent, the best results were observed for populations from Africa, and from East Asia. Those from America were the most challenging, followed by Europe. Also, for these two challenging cases, most Europeans were misclassified as American, and vice versa.

**Table 2:** Confusion matrix for continental-level Ancestry classification (oveall classification rate of 96.75%, 206 SNPs)

Continents	Europe	America	Africa	East Asia	South Asia
Europe	94.06%	3.96%	0.00%	0.00%	1.98%
America	10.94%	89.06%	0.00%	0.00%	0.00%
Africa	0.00%	0.00%	100.00%	0.00%	0.00%
East Asia	0.00%	0.00%	0.00%	100.00%	0.00%
South Asia	1.02%	2.04%	0.00%	0.00%	96.94%

### Pairwise classification between sub-populations

**Fig. 3:**
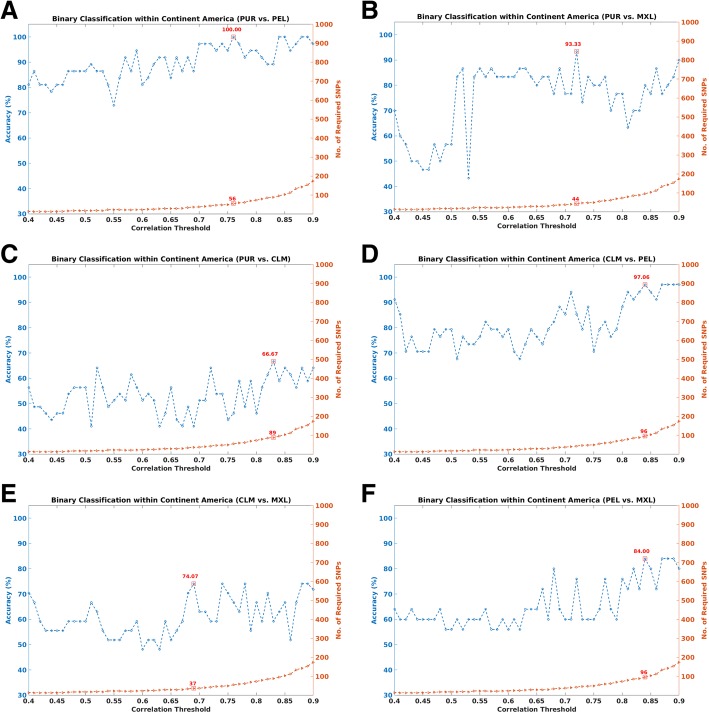
Pairwise classification results with varying correlation thresholds, for subgroups within the continent of America: **a** PUR vs. PEL; **b** PUR vs. MXL, **c** PUR vs. CLM; **d** CLM vs. PEL; **e** CLM vs. MXL; and **f** PEL vs. MXL

Table 3 shows the overall pairwise classification results between sub-populations in each of the five continents in our dataset. The number of SNPs required for each classification have also been noted. From the table, it is evident that in all cases of pairwise classification of closely related populations, we can infer the ethnicity using a small panel of SNPs (less than 200) and for some instances, the accuracy is as high as 100%. For a more detailed analysis, Fig. 3 shows the performance of the proposed methods with increasing correlation thresholds, using sub-populations in the continent of America. As in Fig. 3, the plots for pairwise classification of sub-populations within the continent of America are shown for a range of correlation thresholds *th* = 0.1 to 0.9 with an interval of 0.01. The best performance (#SNPs & accuracy) has been marked with a red square in the figures.

**Table 3:** Results for pairwise/binary classification between sub-populations in each continent

Continent	Sub-populations	Number of SNPs	Correlation Threshold	Accuracy (80–20)
America	PUR-PEL	56	0.76	100.00%
	PUR-MXL	44	0.72	93.33%
	PUR-CLM	89	0.83	66.67%
	CLM-PEL	96	0.84	97.06%
	CLM-MXL	37	0.69	74.07%
	PEL-MXL	96	0.84	84.00%
Europe	GBR-FIN	15	0.47	78.38%
	GBR-IBS	63	0.80	66.67%
	GBR-CEU	30	0.64	67.57%
	GBR-TSI	24	0.61	76.92%
	FIN-IBS	82	0.83	83.33%
	FIN-CEU	130	0.88	80.00%
	FIN-TSI	75	0.82	90.48%
	IBS-CEU	47	0.75	71.43%
	IBS-TSI	82	0.83	77.27%
	CEU-TSI	31	0.67	73.81%
East Asia	CHS-CDX	44	0.73	64.10%
	CHS-KHV	12	0.41	68.29%
	CHS-CHB	30	0.66	64.29%
	CHS-JPT	83	0.84	73.81%
	CDX-KHV	30	0.66	68.42%
	CDX-CHB	120	0.87	76.92%
	CDX-JPT	120	0.87	87.18%
	KHV-CHB	62	0.79	75.61%
	KHV-JPT	92	0.85	82.93%
	CHB-JPT	83	0.84	71.43%
South Asia	PJL-BEB	29	0.65	74.29%
	PJL-STU	57	0.78	62.50%
	PJL-ITU	29	0.65	70.00%
	PJL-GIH	153	0.89	100.00%
	BEB-STU	42	0.72	72.97%
	BEB-ITU	139	0.88	70.27%
	BEB-GIH	113	0.86	100.00%
	STU-ITU	29	0.65	64.29%
	STU-GIH	79	0.82	100.00%
	ITU-GIH	79	0.82	100.00%
Africa	ACB-GWD	47	0.76	76.74%
	ACB-ESN	20	0.56	79.49%
	ACB-MSL	46	0.75	71.43%
	ACB-YRI	43	0.72	80.49%
	ACB-LWK	60	0.79	79.49%
	ACB-ASW	15	0.49	81.48%
	GWD-ESN	46	0.75	77.27%
	GWD-MSL	73	0.82	72.50%
	GWD-YRI	132	0.88	100.00%
	GWD-LWK	132	0.88	100.00%
	GWD-ASW	132	0.88	96.88%
	ESN-MSL	102	0.86	69.44%
	ESL-YRI	132	0.88	100.00%
	ESN-LWK	132	0.88	100.00%
	ESN-ASW	132	0.88	96.43%
	MSL-YRI	38	0.71	100.00%
	MSL-LWK	132	0.88	100.00%
	MSL-ASW	73	0.82	91.67%
	YRI-LWK	28	0.65	78.57%
	YRI-ASW	146	0.89	90.00%
	LWK-ASW	162	0.90	85.71%

As can be observed, it is relatively easy to distinguish between individuals from certain sub-populations, even within the same continent. For instance, Fig. 3a shows that individuals of Porto Rican (PUR) descent are relatively easy to distinguish from those with Peruvian (PEL) descent, achieving a 100% accuracy rate, using 56 SNPs, under our approach. Similarly for Columbia (CLM) and Peru (PEL) (Fig. 3c). As before, accuracy generally increases with increasing correlation thresholds (and hence more SNPs), but this is not monotonic. However, we can also see some challenging cases, such as Columbia (CLM) and Mexico (MXL), (see Fig. 3e), where the highest classification rate is only about 74%, using 37 SNPs. Even increasing the number of SNPs beyond 37 could not improve the result.

### Computation time

The experiments were performed on a personal computer running on Intel Core i7-7700 K Quad-Core 4.2 GHz Desktop Processor, 16GB RAM, with 4 TB 64 MB Cache Hard Drive. Part of the proposed approach required the evaluation of the predictive power of each SNP. Thus, each SNP is used independently to perform ancestry classification. In the proposed methodology, we may notice that a performance matrix was generated before initiating SNP selection for a certain correlation threshold. With the reduced set of 6404 SNPs, the algorithm had to run 6404 times to generate the performance matrix, where each time only one SNP is being used to perform classification. The average time it takes to evaluate the performance of a single SNP is approximately 1.17 s. With 6404 SNPs, the time required to construct the whole performance matrix is about 2 h. By using a graphics processing unit (GPU), we can reduce the total time for generating the performance matrix to 1.5 h. After we generate the performance matrix, the SNP selection process starts. We compute pairwise correlation between SNPs and based on a certain correlation threshold we identify a panel of non redundant (or important) SNPs. The value of the correlation threshold determines the size of the SNP panel and the number of SNP features in a panel determines how much time will be taken by the classifier to perform classification. SNP selection time for continental level classification using correlation threshold 0.9 is approximately 27.35 s, where 184 SNPs have been selected.

### Comparative performance evaluation

We have performed a limited comparison of our proposed approaches with related work. Table 4 shows the comparative performance of our proposed methods on continental-level ancestry classification, when compared with other related methods. Table 5 presents similar comparative performance of our proposed method for binary/pairwise classification of sub-populations against other related methods in the literature. The comparative results show the proposed methods are competitive with the state-of-the-art methods, even when using information from just one chromosome.

**Table 4:** Comparative Performance on continental-level Ancestry classification

Basic Method	Data Size	Datasets Used	Classification Rate (%)
STRUCTURE [36]	664	Mutiple datasets	96.1
SNPforID [4]	2689	1000 Genome, HGDP, NIST	98.8
STRUCTURE [37]	6410	Mutiple datasets	81.4
Random match probability [5]	451	Own collection	77.0 (+ 21.6 thresholded out)
Proposed	2504	1000 Genome Phase 3	99.19 (614 SNPs)
Proposed	2504	1000 Genome Phase 3	96.75 (206 SNPs)

**Table 5:** Comparative Performance In Sub-Population-level Ancestry classification

Pairwise sub-populations	Continent	Method	Data size	Datasets	Classification rate (%)	Number of attributes used
CEU-TSI	europe	ethnopred [38]	267	hapmap iii	86.6 ± 2.4	180 SNPs
CHB-JPT	east asia	ethnopred [38]	250	hapmap iii	95.6 ± 3.9	877 SNPs
LWK-MKK	africa	ethnopred [38]	294	hapmap iii	95.9 ± 1.5	341 SNPs
JPT-CHB	east asia	bayesian [39]	9104	own collection	74.9 (77.2***)	15 STR loci
JPT-KOR	east asia	bayesian [39]	731	own collection	67.9 (63.7)	15 STR loci
CHB-KOR	east asia	bayesian [39]	731	own collection	69.6 (62.4)	15 STR loci
–	europe	proposed	503	1000 genome phase 3	76.6*	58 snps**
–	africa	proposed	661	1000 genome phase 3	87.02*	87 snps**
–	east asia	proposed	504	1000 genome phase 3	73.3*	68 snps**

*Average accuracy of all pairwise sub-population classifications within the given continent

**Average number of SNPs required in all pairwise sub-population classifications within the given continent

***Results obtained without normalization

## Discussion and conclusion

Prediction of continental ancestry from genetic sequences have been studied for years. However, much less has been done on prediction of ancestry for closely-related sub-populations, for instance, those that are within the same country, or continent, especially under resource constraints, with potentially limited or missing genomic data. In this work, we have developed an ancestry identification system to predict the continental origin of an unknown individual and also distinguish between closely related sub-populations within a continent. We used only SNPs from just one chromosome (namely, Chromosome 1) for our analysis, and to identify different panels of ancestry informative SNPs. We have applied both machine learning and statistical techniques to select candidate SNPs. Our results show that one single chromosome (Chromosome 1, in this case), if carefully analyzed, could hold enough information for accurate estimation of human biogeographical ancestry. This has a significant implication in terms of the computational resources required for analysis of ancestry, and in the applications of such analyses, such as in studies of genetic diseases, forensics, and biometrics.

We have essentially considered binary classification, given pairs of sub-populations. Further work can be performed to extend the proposed approach to handle multi-class classification of biogeographical ancestry. Another interesting future work is to investigate the performance of other chromosomes, especially the smaller chromosomes, to see if we can construct equally high-performing panels of AISNPs using an even less amount of data. It will also be interesting to further investigate the identified SNPs to see if there is any connection between them, or their nearby genes, with specific diseases or health problems that are known to be more prevalent in certain geographic regions.

## Abbreviations

AIM: Ancestry informative marker
AISNP: Ancestry informative SNP
DBSCAN: Density-based spatial clustering of applications with noise
DNA: Deoxynucleic acid
GPU: Graphics processing units
GWAS: Genome-wide association studies
PCA: Principal component analysis
SNP: Single nucleotide polymorphisms
STR: Short tandem repeats
