# Correction to: Human ancestry indentification under resource constraints -- what can one chromosome tell us about human biogeographical ancestry?

**DOI:** 10.1186/s12920-018-0464-5

**Published:** 2019-01-09

**Authors:** Tanjin T. Toma, Jeremy M. Dawson, Donald A. Adjeroh

**Affiliations:** Lane Department of Computer Science and Electrical Engineering, West Virginia University, Morgantown, WV USA

**Correction to: BMC Med Genomics**

10.1186/s12920-018-0412-4

After publication of this supplement article [1], it was flagged that there is a typo in the article’s title.

Please be advised that the correct title is “Human ancestry identification under resource constraints -- what can one chromosome tell us about human biogeographical ancestry?”

That is, “indentification” should read “identification”.
